# Whether induction of labor ahead in low-risk women improves pregnancy outcomes?: A retrospective cohort, observational study

**DOI:** 10.1097/MD.0000000000033426

**Published:** 2022-04-07

**Authors:** Huiyan Ren, Qing Zuo, Yi Pan, Xinxin Zhu, Tingting Yin, Min Zhang, Yin Yin, Zhiping Ge, Ziyan Jiang, Hongmei Lu

**Affiliations:** a Department of Obstetrics and Gynaecology, First Affiliated Hospital of Nanjing Medical University, Nanjing, China.

**Keywords:** delivery, induction, labor, outcomes, timing

## Abstract

The appropriate timing of delivery for pregnancies has always been a concern for medical staff, and the timing of elective labor induction at 41 weeks in low-risk pregnant women has always been controversial. We compared maternal and fetal outcomes between gestational age at 40 0/7 to 40 6/7 and 41 0/7 to 41 6/7 weeks. This retrospective cohort study was conducted at the obstetrics department of Jiangsu Province Hospital from January 1^st^ to December 31^st^ in 2020. Maternal medical records and neonatal delivery data were collected. One-way analysis of variance, Mann–Whitney *U* test, χ^2^ test, Fisher exact test and logistig regression analysis were performed. The study included 1569 pregnancies, with 1107 (70.6%) delivered at 40 0/7 to 40 6/7 weeks and 462 (29.4%) delivered at 41 0/7 to 41 6/7 weeks. Intrapartum cesarean section (8% vs 16%, *P* < .001), meconium-stained amniotic fluid (13% vs 19%, *P* = .004), episiotomy (41% vs 49%, *P* = .011), and macrosomia (13% vs 18%, *P* = .026) were significantly lower at 40 0/7 to 40 6/7 weeks. The premature rupture of membranes rate (22% vs 12%, *P* < .001), vaginal delivery rate of artificial rupture of membrane induction (83% vs 71%, *P* = .006) and balloon catheter combined with oxytocin induction (88% vs 79%, *P* = .049) were significantly higher at 40 0/7 to 40 6/7 weeks. Low-risk women who delivered at 40 0/7 to 40 6/7 weeks showed better outcomes in terms of the mother’s and baby’s health, such as decreased rates of intrapartum cesarean section, meconium-stained amniotic fluid, episiotomy, and macrosomia, compared with those who delivered at 41 0/7 to 41 6/7 weeks.

## 1. Introduction

The appropriate timing of delivery for pregnancies has always been a concern of medical staff. Fetal lung maturity gradually increases with gestational age, and premature delivery may lead to neonatal acidemia, whereas late delivery may increase maternal and neonatal risks. Neonates delivered at 36 to 38 weeks after confirmed fetal lung maturity are at higher risk of adverse outcomes than those delivered at 39 to 40 weeks.^[1]^ Pulmonary maturity generally occurs after full-term birth (39 0/7–40 6/7 weeks).^[2]^ Therefore, inducing pregnancy at 39 0/7 to 40 6/7 weeks may be proper from the angle of fetal lung maturity.

At present, elective labor induction is commonly performed after 41 weeks in low-risk women without labor onset.^[3,4]^ However, with increases in gestational weeks, increases are observed in fetal weight, the probability of macrosomia, the rate of cephalopelvic disproportion, and the occurrence of shoulder dystocia, cesarean section, neonatal asphyxia and birth injury.^[5–8]^ Thus, we sought to determine whether inducing low-risk pregnant women in advance at 40 weeks will lead to improved maternal and fetal outcomes. Therefore, the goal of this study was to compare maternal and fetal outcomes between gestational age at 40 0/7 to 40 6/7 and 41 0/7 to 41 6/7 weeks.

## 2. Materials and methods

### 2.1. Study population

This retrospective cohort study assessed data from the obstetrics department of Jiangsu Province Hospital from January 1^st^ to December 31^st^, 2020. These data included all maternal medical records and neonatal deliveries. The inclusion criteria were a low-risk singleton pregnancy delivered at 40 0/7 to 41 6/7 weeks of gestation at our hospital. Low-risk was defined as the absence of any condition that might be a maternal or fetal indication for delivery in a short time, such as any antepartum hypertensive disorder, cardiac disease or renal insufficiency.^[9]^ Cases with electing cesarean delivery, vaginal birth after cesarean section and partial data loss were excluded. We divided pregnancies meeting the inclusion criteria into a full-term group (40 0/7–40 6/7 weeks) and a late-term group (41 0/7–41 6/7 weeks).^[10]^ This study was approved by the Ethics Committee of Jiangsu Province Hospital [Ethics Committee 2020-SR-256].

### 2.2. Outcome measures

#### 1.2.2. Maternal complications.

The main variable exposed in our analysis was gestational age at delivery. The maternal complications were mainly episiotomy, instrumental delivery, intrapartum cesarean section (ICS), postpartum hemorrhage (PPH), shoulder dystocia, obstetric anal sphincter injury, meconium-stained amniotic fluid (MSAF), intrapartum cervical laceration, epidural analgesia, amniotic fluid embolism, and labor progression duration. An episiotomy performed at the time of crowning was defined as a surgical incision made to widen the vaginal opening for the delivery of the fetus.^[11]^ Instrumental delivery was defined as vacuum extraction or forceps delivery.^[12]^ In our hospital, it mainly referred to vacuum extraction. PPH was defined as blood loss in excess of 500 mL after vaginal delivery and in excess of 1000 mL after cesarean delivery in the first 24 hours after labor, and it was divided into mild PPH (estimated blood loss > 500 mL) and severe PPH (estimated blood loss > 1000 mL).^[13]^ The total amount of blood loss was measured by weighing soaked materials and by use of the suction system and collector bags in the operating room. Shoulder dystocia was defined as the inability of the fetus to be delivered by traditional midwifery methods when the symphysis pubis obstructed the anterior descending of the shoulder or the posterior shoulder of the fetus was impacted on the maternal sacral promontory.^[14]^ obstetric anal sphincter injury, including 3rd- and 4th-degree vaginal tears, damages the anal sphincter complex, and anorectal mucosa.^[15]^ Intrapartum cervical laceration was defined as laceration with abnormal vaginal bleeding or requiring cervical suturing.^[16]^ Amniotic fluid embolism was defined as the abnormal activation of proinflammatory mediator systems triggered by entrance into the maternal circulation of material from the fetal compartment.^[17]^ The first stage of labor was defined as beginning from maternal perception of regular contractions to dilation. The second stage of labor was defined as the time from full dilation to delivery of the neonate.^[3]^

### 2.3. Neonatal complications

The neonatal complications were mainly macrosomia, neonatal intensive care unit (NICU) admission, 5-minute Apgar score ≤ 7, neonatal acidemia, subgaleal hemorrhage, glycopenia, neonatal jaundice, digestive syndrome, and clavicle fracture. Neonatal acidemia manifested as dyspnea, shallow and rapid breathing, moaning, and low pulse oxygen. We also diagnosed neonatal acidemia for umbilical artery pH < 7.1, chest X-ray abnormal and white blood cells > 20*10^9/L.^[18]^ Hypoglycaemia was defined as a neonatal blood glucose < 2.5 mmol/L.^[19]^ A pediatric professional doctor diagnosed neonatal jaundice requiring treatment through clinical examination and bilirubin levels due to the different peak serum bilirubin levels of neonates of different races.

### 2.4. Statistical analysis

All statistical analyzes were conducted using Excel and SPSS 26.0. Qualitative data were expressed as percentages (%), differences between groups were assessed by χ^2^ test, and Fisher exact test was used to test significance. Normally distributed data were expressed as the mean ± standard deviation, and differences between groups were analyzed by 1-way analysis of variance. Nonnormally distributed data were expressed as the median (minimum, maximum), and differences between groups were tested using the Mann–Whitney *U* test. Logistic regression analysis was performed on the related factors that might be with statistical significance. Statistical significance was reached at *P* < .05.

## 3. Results

### 3.1. Study population

**Figure 1.**
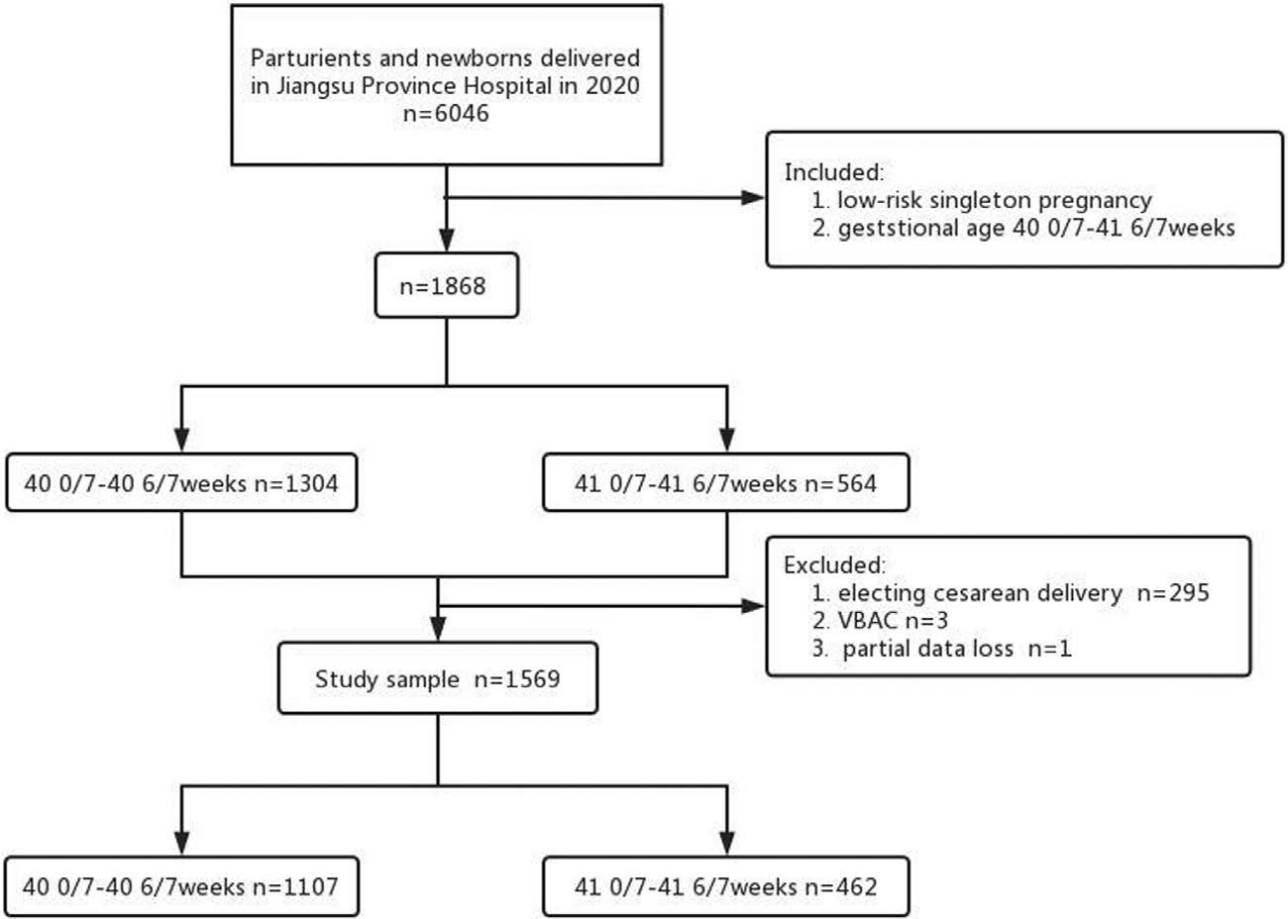
Flow diagram of study selection process. Full-term group was defined as 40 0/7–40 6/7 weeks of delivery and late-term group was defined as 41 0/7–41 6/7 weeks of delivery. VBAC = vaginal birth after cesarean section.

In 2020, a total of 6046 women were booked for delivery in our hospital and 1868 low-risk singleton deliveries at 40 0/7 to 41 6/7 weeks were included in the study. Of these, 299 pregnancies were excluded based on the following factors: elected cesarean delivery (n = 295), vaginal birth after cesarean section (n = 3) and partial data loss (n = 1). Ultimately, a total of 1569 pregnancies met the inclusion criteria. Among them, 1107 (71%) pregnancies delivered at 40 0/7 to 40 6/7 weeks and 462 (29%) delivered at 41 0/7 to 41 6/7 weeks (Fig. 1).

Maternal characteristics in the study groups are compared in Table 1. A total of 1262 (approximately 80%) were nulliparous, and 73 (<5%) were assisted reproductive pregnancies. Approximately 60% of women had complications, including anemia, hypothyroidism, diabetes, and other unusual complications. Statistical significance was not observed in the cases of anemia or hypothyroidism between the 2 groups. The risk of premature rupture of membranes (PROM) in the full-term group was significantly higher than that in the late-term group (22% vs 12%, *P* < .001). The prevalence rate of gestational diabetes mellitus (GDM) in the full-term group was significantly higher than that in the late-term group (16% vs 8%, *P* < .001).

**Table 1:** Maternal baseline characteristics in 2 groups.

Baseline characteristics	40 0/7–40 6/7 w	41 0/7–41 6/7 w	*P* value	T/X^2^
	N = 1107	N = 462		
Maternal age/yr	29.3 ± 3.5	29.0 ± 3.6	.087†	1.72
Maternal body mass index (kg/m^2^)	27.3 ± 6.3	27.4 ± 3.0	.865†	−0.17
Nulliparous–no. (%)	888 (80)	374 (81)	.738	0.11
Gestational age/w–median (min, max)	40.43 (40–40.86)	41.14 (41–41.86)	.000‡	-
Gravidity–median (min, max)	1 (1–6)	1 (1–6)	.595‡	-
Parity–median (min, max)	1 (1–4)	1 (1–3)	.694‡	-
Assisted reproduction–no. (%)	57 (5)	16 (3)	.148	2.09
Complications–no. (%)	637 (58)	261 (56)	.702	0.15
GDM–no. (%)	172 (16)	37 (8)	.000*	16.00
Hypothyroidism–no. (%)	221 (20)	107 (23)	.156	2.01
Anemia–no. (%)	99 (9)	40 (9)	.856	0.03
PROM–no. (%)	243 (22)	56 (12)	.000*	20.42

GDM = gestational diabetes mellitus, PROM = premature rupture of membranes.

* Mesns significant difference between groups.

† one-way analysis of variance.

‡ Mann-Whitney *U* test.

Different modes of labor are compared in Table 2. The spontaneous labor rate was significantly higher than that in the late-term group (54% vs 20%, *P* < .001). The full-term group was lower than the late-term group in terms of the rate of induction of balloon catheter combined with oxytocin (BCCO) (9% vs 46%, *P* < .001), the rate of oxytocin induction (22% vs 27%, *P* = .012) and artificial rupture of membrane (AROM) induction (21% vs 31%, *P* < .001). The bishop score of parturient with BCCO induction was not statistically significant (*P* = .055). However, the late-term group had a higher final vaginal delivery rate of BCCO (88% vs 79%, *P* = .049) and AROM (83% vs 71%, *P* = .006). Significant differences were not observed in the vaginal delivery rate of oxytocin induction (80% vs 76%, *P* = .431).

**Table 2:** Comparison of different mode of labor.

Mode	40 0/7–40 6/7w	41 0/7–41 6/7w	*P* value	T/X^2^
	N = 1107	N = 462		
Spontaneous labor–no. (%)	597 (54)	94 (20)	.000*	149.17
BCCO–no. (%)	100 (9)	212 (46)	.000*	277.90
Bishop score	3.07 ± 1.30	2.77 ± 1.28	.055	1.93
Vaginal delivery–no. (%)	88 (88)	167 (79)	.049*	3.87
Oxytocin–no. (%)	239 (22)	127 (27)	.012*	6.34
Vaginal delivery–no. (%)	191 (80)	97 (76)	.431	0.62
AROM–no. (%)	238 (21)	143 (31)	.000*	15.84
Vaginal delivery–no. (%)	198 (83)	102 (71)	.006*	7.51

AROM = artificial rupture of membranes, BCCO = balloon catheter combined with oxytocin.

* Mesns significant difference between groups.

Compared with the induction group, the spontaneous labor group had a lower body mass index (OR 0.96; 95% CI 0.92–0.99), a lower gestational age (OR 0.23; 95% CI 0.18–0.30), a lower epidural analgesia rate (OR 0.56; 95% CI 0.43–0.73), a lower neonatal birth weight (OR 1.000; 95% CI 0.999–1.000), a higher maternal height (OR 1.03; 95% CI 1.01–1.06), a higher amniotic fluid pollution grade (OR 0.89; 95% CI 0.81–0.98), and more parity times (OR 1.96; 95% CI 1.39–2.77). The gravidity times showed no difference between the 2 groups, as shown in Tables 3 and 4.

**Table 3:** Comparison of indiction group and spontaneous labor group.

Baseline characteristics	indiction	spontaneous labor	*P* value
	N = 878	N = 691	
Maternal age/yr	29.22 ± 3.56	29.21 ± 3.47	.989
Maternal body mass index (kg/m2)	27.72 ± 6.84	26.89 ± 3.24	.003*
Maternal height/cm	162.02 ± 5.99	162.66 ± 4.89	.022
Gestational age/w	40.78 ± 0.50	40.47 ± 3.67	.000*
Gravidity–median (min, max)	1 (1–6)	1 (1–6)	.000*
Parity–median (min, max)	1 (1–4)	1 (1–3)	.000*
Epidural analgesia			.000*
Yes	730 (83)	486 (70)	
No	148 (17)	205 (30)	
Amniotic fluid			
Clear	560 (64)	493 (71)	.002*
Grade I	61 (7)	55 (8)	.189
Grade II	99 (11)	68 (10)	
Grade III	158 (18)	75 (11)	.000*
Neonatal birth weight/g	3608.13 ± 368.67	3531.79 ± 370.79	.000*

* Mesns significant difference between groups.

**Table 4:** Logistic regression model assessing indiction group vs spontaneous labor group.

Variables	B	S.E.	Wald	Sig.	OR (95% CI)
Maternal body mass index (kg/m^2^)	−0.043	0.018	5.842	0.016*	0.957 (0.924–0.992)
Maternal height/cm	0.032	0.012	7.239	0.007*	1.033 (1.009–1.057)
Gestational age/w	−1.474	0.128	132.205	0.000*	0.229 (0.178–0.295)
Gravidity	−0.023	0.077	0.087	0.769	0.978 (0.840–1.137)
Parity	0.675	0.176	14.734	0.000*	1.964 (1.391–2.773)
Epidural analgesia	−0.58	0.137	17.834	0.000*	0.560 (0.428–0.733)
Amniotic fluid	−0.115	0.050	5.305	0.021*	0.891 (0.808–0.983)
Neonatal birth weight/g	0.000	0.000	8.267	0.004*	1.000 (0.999–1.000)
Constant	57.005	5.506	107.191	0.000*	

* Mesns significant difference between groups.

### 3.2. Maternal outcomes

Maternal outcomes in the study groups are compared in Table 5. The successful vaginal delivery rate in the full-term group was significantly higher than that in the late-term group (90% vs 80%, *P* < .001), and the rate of episiotomy was also significantly decreased (41% vs 49%, *P* = .011). The risk of ICS in the full-term group was significantly lower than that in the late-term group (8% vs 16%, *P* < .001). Significant differences were not observed between the full-term and late-term groups in cephalopelvic disproportion (6% vs 10%, *P* = .073) or fetal distress (29% vs 39%, *P* = .133). The rate of MSAF in the late-term group was significantly higher than that in the full-term group (19% vs 13%, *P* = .004). The epidural analgesia rate during labor in the full-term group was lower than that in the late-term group (76% vs 81%, *P* = .034). Significant differences were not observed in labor duration, postpartum hemorrhage, shoulder dystocia, or intrapartum cervical laceration rates between the 2 groups.

**Table 5:** Maternal and perinatal outcomes in 2 groups.

Outcomes	40 0/7–40 6/7 w	41 0/7–41 6/7 w	*P* value	T/X^2^
	N = 1107	N = 462		
Vaginal delivery–no. (%)	999 (90)	369 (80)	.000*	31.41
Instrumental delivery–no. (%)	9 (0.8)	7 (1.9)	.216	1.53
Episiotomy–no. (%)	408 (41)	179 (49)	.011*	6.47
ICS–no./total no. (%)	108/1304 (8)	93/564 (16)	.000*	27.62
Cephalopelvic disproportion–no. (%)	67 (6)	46 (10)	.073	3.21
Fetal distress–no. (%)	31 (29)	36 (39)	.133	2.25
Postpartum hemorrhage/mL–no. (%)	158 (14)	77 (17)	.226	1.47
500–999–no. (%)	113 (10)	52 (11)	.538	0.38
≥1000–no. (%)	45 (4)	25 (5)	.239	1.39
Epidural analgesia–no. (%)	842 (76)	374 (81)	.034*	4.47
Shoulder dystocia–no. (%)	4 (0.4)	1 (0.2)	1.000	0.00
MSAF–no. (%)	146 (13)	87 (19)	.004*	8.21
OASIS–no. (%)	0	0	-	
Intrapartum cervical laceration–no. (%)	5 (0.5)	2 (0.4)	1.000	0.00
Amniotic fluid embolism–no. (%)	1 (0.1)	0	1.000	0.00
Labor duration in nullipara/h				
The first stage (analgesia)	10.4 ± 3.4	10.9 ± 3.2	.054†	−1.93
The second stage (analgesia)	1.3 ± 0.7	1.3 ± 0.8	.107†	−1.61
The first stage (non-analgesia)	7.9 ± 3.5	8.6 ± 3.7	.275†	−1.10
The second stage (non-analgesia)	1.0 ± 0.6	1.1 ± 0.6	.365†	−0.91
Labor duration in multipara/h				
The first stage (analgesia)	6.2 ± 2.5	6.6 ± 2.9	.335†	−0.97
The second stage (analgesia)	0.5 ± 0.4	0.5 ± 0.4	.368†	−0.90
The first stage (non-analgesia)	4.5 ± 1.9	5.0 ± 2.3	.228†	−1.21
The second stage (non-analgesia)	0.3 ± 0.3	0.5 ± 0.5	.097†	−1.70
Neonatal birth weight/g	3548.6 ± 374.5	3636.6 ± 356.7	.000*,†	−4.3
Macrosomia–no. (%)	146 (13)	81 (18)	.026*	4.97
4000 g–4499 g–no. (%)	135 (12)	74 (16)	.042*	4.12
≥4500g–no. (%)	11 (1)	7 (1.5)	.377	0.78
Male newborn–no. (%)	563 (51)	234 (51)	.940	0.01
NICU admission–no. (%)	175 (16)	90 (19)	.077	3.13
1 minute Apgar ≤ 7–no. (%)	10 (0.9)	2 (0.4)	.511	0.43
Neonatal acidemia–no. (%)	81 (7)	42 (9)	.233	1.42
Subgaleal hemorrhage–no. (%)	3 (0.3)	1 (0.2)	1.000	0.00
Glycopenia–no. (%)	3 (0.3)	1 (0.2)	1.000	0.00
Neonatal jaundice–no. (%)	51 (5)	23 (5)	.752	0.10
Digestive syndrome–no. (%)	14 (1)	5 (1)	.763	0.09
Clavicle fracture–no. (%)	1 (0.1)	1 (0.2)	1.000	0.00

ICS = intrapartum cesarean section, MSAF = meconium-stained amniotic fluid, OASIS = obstetric anal sphincter injury, NICU = neonatal intensive care unit.

* Mesns significant difference between groups.

† one-way analysis of variance.

After controlling and adjusting for the influence of confounding factors, compared with the late-term group, the full-term group had a higher risk of PROM (OR 3.25; 95% CI 2.18–4.84), GDM (OR 4.15; 95% CI 2.67–6.44) and vaginal delivery (OR 1.64; 95% CI 1.13–2.39) and a lower balloon catheter combined with oxytocin induction rate (OR 0.05, 95% CI 0.03–0.09), a lower oxytocin induction rate (OR 0.16; 95%CI 0.09–0.28). The maternal age, spontaneous labor rate, epidural analgesia rate, MSAF rate, NICU admission rate and AROM rate showed no difference between the 2 groups, as shown in Table 6.

**Table 6:** Logistic regression model assessing full-term group vs late-term group.

Variables	B	S.E.	Wald	Sig.	OR (95% CI)
Maternal age/yr	0.031	0.019	2.745	0.098	1.031 (0.994–1.070)
GDM	1.423	0.225	40.086	0.000*	4.148 (2.671–6.444)
PROM	1.177	0.204	33.24	0.000*	3.246 (2.175–4.843)
spontaneous labor	−0.281	0.31	0.818	0.366	0.755 (0.411–1.387)
Vaginal delivery	0.497	0.191	6.78	0.009*	1.644 (1.131–2.391)
Epidural analgesia	−0.055	0.164	0.114	0.736	0.946 (0.686–1.305)
MSAF	−0.351	0.183	3.679	0.055	0.704 (0.492–1.008)
Neonatal birth weight/g	−0.001	0.000	9.402	0.002*	0.999 (0.999–1.000)
NICU	0.015	0.176	0.007	0.931	1.015 (0.719–1.433)
BCCO	−2.941	0.282	108.508	0.000*	0.053 (0.030–0.092)
Oxytocin	−1.815	0.282	41.291	0.000*	0.163 (0.094–0.283)
AROM	−0.099	0.21	0.224	0.636	0.905 (0.600–1.367)
Constant	2.562	0.941	7.408	0.006*	12.963

AROM = artificial rupture of membranes, GDM = gestational diabetes mellitus, BCCO = balloon catheter combined with oxytocin, MSAF = meconium-stained amniotic fluid, NICU = neonatal intensive care unit, PROM = premature rupture of membranes.

* Means significant difference between groups.

### 3.3. Neonatal outcomes

Neonatal outcomes in the study groups are compared in Table 3. The average neonatal birth weight of the late-term group was higher than that of the full-term group (*P* < .001), and the macrosomia rate was also significantly higher than that of the full-term group (18% vs 13%, *P* = .026). The main difference was in the range of 4000 to 4499 g (16% vs 12%, *P* = .042). Significant differences were not observed in the rates of NICU admission, 5-minute Apgar score ≤ 7, neonatal acidemia, subgaleal haemorrhage, glycopenia, neonatal jaundice, swallowing syndrome or clavicle fracture between the 2 groups. Compared with the the late-term group, full-term group had the lower neonatal birth weight (OR 0.999, 95% CI 0.999–1.000), as shown in Table 6.

## 4. Discussions

At present, the timing of delivery is controversial, and many studies are exploring more suitable timings. Studies have shown that elective induction before 39 weeks increases the rate of NICU admission, prolongs the neonatal hospitalization time, results in a high readmission rate within 2 weeks after delivery, and increases emergency department visits.^[20,21]^ A large multicenter RCT along with some retrospective studies showed that elective induction at 39 weeks reduced the cesarean section rate, pregnancy hypertension risk, perinatal infection, neonatal adverse perinatal outcomes (respiratory complications, NICU admission, perinatal death) and did not affect neonatal early literacy and numeracy ability.^[9,22,23]^ Therefore, elective induction after 39 weeks in low-risk nulliparous women is recommended. However, there is little evidence for elective induction between 40 0/7 and 40 6/7 weeks.

Nowadays, elective labor induction is commonly recommended after 41 weeks in low-risk women without labor onset. The reason for inducing until 41 weeks is to wait for the maturity of the cervix and to have higher chances of spontaneous labor and PROM. However, our study showed no difference in the bishop scores in the 2 groups and less opportunity for spontaneous labor and PROM after 41 weeks. If we need to induce these women by AROM or balloon catheter, then there is a higher rate for vaginal delivery at 40 0/7 to 40 6/7 weeks. Furthermore, we also found decreased ICS, meconium-stained amniotic fluid, episiotomy and macrosomia rates at 40 0/7 to 40 6/7 weeks. Studies have shown that the incidence of MSAF increases with gestational age,^[24,25]^ which is consistent with our findings. Therefore, our study indicated that waiting until 41 weeks in low-risk women was not necessarily beneficial; however, our study is retrospective and needs to be further proven by prospective studies.

An international cohort study showed that after 39 weeks, the risk of PPH increased with gestational age.^[26]^ Our results did not reflect this obvious difference, although preliminarily observations indicated that the rate of PPH in the late-term group was slightly greater than that in the full-term group (17% vs 14%, *P* = .226). If we increased the amount of data, statistical significance might be obtained. In our study, the prevalence rate of GDM in the full-term group was significantly higher than that in the late-term group. This might be because ACOG recommended that for women with GDM that was controlled with only diet and exercise, it was appropriate to control the gestation period within 40 6/7 weeks.^[27]^ Those persisted beyond 41 weeks were due to lack of medical compliance. There was 1 case of amniotic fluid embolism in our study, which occurred during spontaneous vaginal delivery at 40 3/7 weeks. One hour after delivery, the patient experienced dyspnea, a sudden drop in blood pressure (86/42 mm Hg) and oxygen saturation (80%). After a series of rescue measures, such as open veins, oxygen inhalation, blood pressure boosting, and anti-allergic measures, the final prognosis was good.

Our study indicated that the macrosomia rate was higher at 41 0/7 to 41 6/7 weeks. No differences in adverse neonatal outcomes were noted in either group, which is consistent with a previous study.^[21]^ Many studies have revealed the risks of macrosomia, such as shoulder dystocia, clavicle fractures, breathing problems, decreased 5-minute Apgar score, hypoglycemia, meconium aspiration, and more.^[5–8]^ From this point of view, it is a wise choice to induce at 40 weeks to reduce the risk of macrosomia. However, our conclusion is not to overrule induction timing after 41 weeks. We found that PPH and NICU admission did not increase due to expectations; therefore, induction after 41 weeks cannot be considered inappropriate. For low-risk pregnant women without suspicious macrosomia, we can expect up to 41 weeks according to their willingness. Cavoretto’s studies showed that longer labor duration^[28]^ and higher meconium-stained amniotic fluid rate^[29]^ could increase the risk of neonatal acidemia. In our study, there were no differences in labor duration and neonatal acidemia between the 2 groups, which was consistent with previous studies. However the MSAF rate decreased at at 40 0/7 to 40 6/7 weeks in our study, which seemed different from previous studies, and this might be due to the limited sample size.

## 5. Strengths and limitations

In China, elective labor induction is recommended after 41 weeks in low-risk women. Our study showed that induction of labor ahead might improve the pregnancy outcomes of these women. We believe our study may promote the development and research of relevant guidelines in China. Meanwhile, the limitations of our study should also be noted. This study was a single-center retrospective study, which was small in scale and some data records were easy to be missing, There are many factors affecting delivery, however the indicators included in this article could not cover all. Follow-up researches may need to increase the sample size and relevant observation indicators for further research to provide reference basis for clinical treatment plans.

## 6. Conclusions

In conclusion, deliveries at 40 0/7 to 40 6/7 weeks had better outcomes for low-risk mothers and their babies, such as decreased rates of ICS, meconium-stained amniotic fluid, episiotomy, and macrosomia, compared with deliveries at 41 0/7 to 41 6/7 weeks. From our results, we suppose that elective induction at 40 weeks of gestation might be superior to that at 41 weeks in terms of certain maternal and neonatal outcomes. However, in clinical practice, the appropriate timing of delivery should be discussed and choose a personalized delivery plan. Moreover, the conclusions remain to be further proven by prospective studies.

## Acknowledgements

We thank all authors participating in this study.

## Author contributions

**Conceptualization:** Zhiping Ge, Hongmei Lu.

**Data curation:** Huiyan Ren, Qing Zuo, Xinxin Zhu, Min Zhang, Yin Yin.

**Formal analysis:** Qing Zuo, Tingting Yin.

**Funding acquisition:** Qing Zuo, Zhiping Ge, Hongmei Lu, Jiang ziyan.

**Investigation:** Qing Zuo, Min Zhang.

**Resources:** Yi Pan, Min Zhang.

**Software:** Yin Yin.

**Validation:** Tingting Yin, Yin Yin.

**Writing – original draft:** Huiyan Ren.

**Writing – review & editing:** Yi Pan, Zhiping Ge, Hongmei Lu, Jiang ziyan

## References

[1] Bates E Rouse DJ Mann ML. Neonatal outcomes after demonstrated fetal lung maturity before 39 weeks of gestation. Obstet Gynecol. 2010;116:1288–95. 21099593 10.1097/AOG.0b013e3181fb7ece PMC4074509

[2] Tita ATN Jablonski KA Bailit JL. Neonatal outcomes of elective early-term births after demonstrated fetal lung maturity. Am J Obstet Gynecol. 2018;219:296.e1–8. 10.1016/j.ajog.2018.05.011 PMC6143365 29800541

[3] Caughey AB Cahill AG Guise JM Safe prevention of the primary cesarean delivery. Am J Obstet Gynecol. 2014;210:179–93. 24565430 10.1016/j.ajog.2014.01.026

[4] Pfützenreuter GR Cavalieri JC Fragoso APO. Factors associated with intrapartum cesarean section in women submitted to labor induction. Rev Bras Ginecol Obstet. 2019;41:363–70. 31247664 10.1055/s-0039-1688966 PMC10316804

[5] Macrosomia: ACOG Practice Bulletin, Number 216. Obstet Gynecol. 2020;135:e18–35. 31856124 10.1097/AOG.0000000000003606

[6] Beta J Khan N Fiolna M. Maternal and neonatal complications of fetal macrosomia: cohort study. Ultrasound Obstet Gynecol. 2019;54:319–25. 30938000 10.1002/uog.20278

[7] Turkmen S Johansson S Dahmoun M. Foetal macrosomia and foetal-maternal outcomes at birth. J Pregnancy. 2018;2018:4790136. 30174954 10.1155/2018/4790136 PMC6106949

[8] Salihu HM Dongarwar D King LM. Phenotypes of fetal macrosomia and risk of stillbirth among term deliveries over the previous four decades. Birth. 2020;47:202–10. 31925852 10.1111/birt.12479

[9] Grobman WA Rice MM Reddy UM. Labor induction versus expectant management in low-risk nulliparous women. N Engl J Med. 2018;379:513–23. 30089070 10.1056/NEJMoa1800566 PMC6186292

[10] Nicholson JM Kellar LC Ural S. New definition of term pregnancy. JAMA. 2013;310:1985–6. 10.1001/jama.2013.277993 24219957

[11] Jha SE. necessity or negligence?. BJOG. 2020;127:1408. 32419358 10.1111/1471-0528.16272

[12] Hankins GD Rowe TF. Operative vaginal delivery–year 2000. Am J Obstet Gynecol. 1996;175:275–82. 8765242 10.1016/s0002-9378(96)70135-7

[13] Leduc D Senikas V Lalonde AB. No. 235-active management of the third stage of labour: prevention and treatment of postpartum hemorrhage. J Obstet Gynaecol Can. 2018;40:e841–55. 30527079 10.1016/j.jogc.2018.09.024

[14] Practice bulletin no 178: shoulder dystocia. Obstet Gynecol. 2017;129:e123–33. 28426618 10.1097/AOG.0000000000002043

[15] Harvey MA Pierce M Alter JE. Obstetrical anal sphincter injuries (OASIS): prevention, recognition, and repair. J Obstet Gynaecol Can. 2015;37:1131–48. 26637088 10.1016/s1701-2163(16)30081-0

[16] Melamed N Ben-Haroush A Chen R. Intrapartum cervical lacerations: characteristics, risk factors, and effects on subsequent pregnancies. Am J Obstet Gynecol. 2009;200:388.e1–4. 10.1016/j.ajog.2008.10.034 19200938

[17] Pacheco LD Saade G Hankins GD. Amniotic fluid embolism: diagnosis and management. Am J Obstet Gynecol. 2016;215:B16–24. 26987420 10.1016/j.ajog.2016.03.012

[18] Hermansen CL Mahajan A. Newborn respiratory distress. Am Fam Physician. 2015;92:994–1002. 26760414

[19] Thompson-Branch A Havranek T. Neonatal hypoglycemia. Pediatr Rev. 2017;38:147–57. 28364046 10.1542/pir.2016-0063

[20] Clark SL Miller DD Belfort MA. Neonatal and maternal outcomes associated with elective term delivery. Am J Obstet Gynecol. 2009;200:156.e1–4. 10.1016/j.ajog.2008.08.068 19110225

[21] Dietz PM Rizzo JH England LJ. Early term delivery and health care utilization in the first year of life. J Pediatr. 2012;161:234–9.e1. 22421263 10.1016/j.jpeds.2012.02.005

[22] Yisma E Mol BW Lynch JW. Elective labor induction vs expectant management of pregnant women at term and children’s educational outcomes at 8 years of age. Ultrasound Obstet Gynecol. 2021;58:99–104. 33030765 10.1002/uog.23141

[23] Grobman WA Caughey AB. Elective induction of labor at 39 weeks compared with expectant management: a meta-analysis of cohort studies. Am J Obstet Gynecol. 2019;221:304–10. 30817905 10.1016/j.ajog.2019.02.046

[24] Ahanya SN Lakshmanan J Morgan BL. Meconium passage in utero: mechanisms, consequences, and management. Obstet Gynecol Surv. 2005;60:45–56. 15618919 10.1097/01.ogx.0000149659.89530.c2

[25] Monen L Hasaart TH Kuppens SM. The aetiology of meconium-stained amniotic fluid: pathologic hypoxia or physiologic foetal ripening? (Review). Early Hum Dev. 2014;90:325–8. 24794302 10.1016/j.earlhumdev.2014.04.003

[26] Butwick AJ Liu C Guo N. Association of gestational age with postpartum hemorrhage: an international cohort study. Anesthesiology. 2021;134:874–86. 33760074 10.1097/ALN.0000000000003730

[27] ACOG practice bulletin no. 190: gestational diabetes mellitus. Obstet Gynecol. 2018;131:e49–64. 29370047 10.1097/AOG.0000000000002501

[28] Cavoretto PI Seidenari A Amodeo S. Quantification of posterior risk related to intrapartum FIGO 2015 criteria for cardiotocography in the second stage of labor. Fetal Diagn Ther. 2021;48:149–57. 33508830 10.1159/000512658

[29] Cavoretto PI Seidenari A Farina A. Hazard and cumulative incidence of umbilical cord metabolic acidemia at birth in fetuses experiencing the second stage of labor and pathologic intrapartum fetal heart rate requiring expedited delivery. Arch Gynecol Obstet. 2023;307:1225–32. 35596749 10.1007/s00404-022-06594-1 PMC10023766

